# Public Health Services Utilization and Its Determinants among Internal Migrants in China: Evidence from a Nationally Representative Survey

**DOI:** 10.3390/ijerph14091002

**Published:** 2017-09-01

**Authors:** Jingya Zhang, Senlin Lin, Di Liang, Yi Qian, Donglan Zhang, Zhiyuan Hou

**Affiliations:** 1 Department of Social Medicine, School of Public Health, National Key Laboratory of Health Technology Assessment (National Health and Family Planning Commission), Collaborative Innovation Center of Social Risks Governance in Health, Fudan University, 138 Yi Xue Yuan Road, Shanghai 200032, China; 15211020069@fudan.edu.cn (J.Z.); 15211020033@fudan.edu.cn (S.L.); 13111020034@fudan.edu.cn (Y.Q.); 2 Department of Health Policy and Management, University of California, Los Angeles, CA 90095, USA; liangdi1988@gmail.com; 3 Department of Health Policy and Management, College of Public Health, University of Georgia, 100 Foster Road, Wright Hall, Athens, GA 30602, USA; dzhang@uga.edu

**Keywords:** migrant, public health, health education, health records, China

## Abstract

There have been obstacles for internal migrants in China in accessing local public health services for some time. This study aimed to estimate the utilization of local public health services and its determinants among internal migrants. Data were from the 2014 and 2015 nationally representative cross-sectional survey of internal migrants in China. Multivariate logistic regressions were used to estimate the relationship between socioeconomic, migration, and demographic characteristics and public health services utilization. Our results showed that internal migrants in more developed eastern regions used less public health services. Those with higher socioeconomic status were more likely to use public health services. The years of living in the city of residence were positively associated with the utilization of public health services. Compared to migration within the city, migration across provinces significantly reduced the probability of using health records (OR = 0.88, 95% CI: 0.86–0.90), health education (OR = 0.97, 95% CI: 0.94–1.00), and health education on non–communicable diseases (OR = 0.92, 95% CI: 0.89–0.95) or through the Internet (OR = 0.96, 95% CI: 0.94–0.99). This study concludes that public health services coverage for internal migrants has seen great improvement due to government subsidies. Internal migrants with lower socioeconomic status and across provinces need to be targeted. More attention should be given to the local government in the developed eastern regions in order to narrow the regional gaps.

## 1. Introduction

During the last three decades, the number of internal migrants has been increasing dramatically in China, reaching 245 million and accounting for 18% of the total population in 2016 [1]. In China, internal migrants (i.e., the floating population) are defined as those who live outside their cities of Hukou registration. Hukou, a permanent household registration system, ties citizens’ access to services according to their Hukou location and Hukou classification (rural vs. urban) [2]. Many public policies and social welfare programs are implemented based on the long-established Hukou system [3,4]. The transfer of Hukou status from rural into urban or from one place to another place is also difficult for citizens when people migrate [5]. Thus, the social welfare and healthcare benefits in urban areas are only available to local residents with registered Hukou, but not to the internal migrants [6]. The health of internal migrants has not been a focus of the urban healthcare system for a long time. The local governments in urban areas have mainly addressed three health problems for internal migrants: infectious diseases, reproductive health, and occupational disease, because these problems have external effects on local residents [3]. However, the prevention and control of non-communicable diseases (NCD) among internal migrants has not become the top priority. 

The national program of Equalization of Essential Public Health Services is one of the very few programs that cover the NCD prevention and control for internal migrants [7]. In 2009, the government commenced this program, with the aim of providing essential public health services for all residents free of charge [8]. The essential public health services included the establishment of health records, health education, immunization, children and maternal health management, and NCD management [8,9]. Government subsidies were given to primary care providers for delivering these services. The government raised the subsidy from 15 renminbi (RMB) per capita in 2009 to 40 RMB in 2015 for financing essential public health services [9].

Unfortunately, migrants can have obstacles in accessing local public health services because these capitation-based subsidies are generally allocated based on the number of residents with local Hukou. Although establishing health records was necessary for disease prevention and treatment, only 23.8% of migrants had established health records in 2013 [10]. While 64.12% of migrants received some health education, more than half of them did not receive any health education on occupational safety and health protection in 2013 [10]. Recently, some cities issued policies to promote the equalization of essential public health services between migrants and local residents. However, previous studies showed that migrants were still less likely to receive public health services than local residents [11]. A survey in Guangdong province showed that in 2014 the rate of utilizing essential public health services was only 30.37% among the migrants, far below that in the urban residents (43.23%) [12].

In fact, little has been known about national status, regional variations, and the determinants of public health services utilization among internal migrants in China. The existing literature has mainly focused on utilization of medical services for internal migrants [13,14]. The few studies that have investigated the utilization of public health services were limited in generalizability because they only focused on several megacities such as Beijing, Shanghai, and Guangzhou [15,16], or focused on patients with certain diseases [17]. Moreover, they emphasized the demographic and socioeconomic factors that affected the utilization of public health services but did not examine the influence of migration characteristics [18]. To provide evidence to achieve the equalization of public health services between residents and migrants, this study aims to investigate the national status, regional variation, and influencing factors of the public health services utilization among Chinese internal migrants by using data from a nationally representative survey for migrants. 

## 2. Materials and Methods

### 2.1. Data and Study Design

The data used in this study were from the 2014 and 2015 waves of the National Internal Migrants Dynamic Monitoring Survey (NIMDMS), conducted by the National Health and Family Planning Commission of China. The NIMDMS is an open access and nationally representative cross-sectional survey of internal migrants aged 15–59 years who did not have the local “Hukou” and had been living in local cities for more than one month.

The sample was drawn using the stratified multi-stage random sampling method with the Probability Proportional to Size (PPS) approach. The survey covered 348 cities from all 32 provincial units in China. Within each city, neighborhoods in urban or suburban areas were randomly selected using the PPS, resulting in a total of 10,300 communities nationwide. In each selected neighborhood, 20 migrants were randomly selected to participate in the survey, and the survey was conducted through face-to-face interviews. Finally, there were 200,937 and 206,000 migrants who participated in the survey in 2014 and 2015, respectively. All subjects gave their informed consent for inclusion before they participated in the study. 

The questionnaires included five sections: (I) basic demographic characteristics including gender, age, marital status, etc.; (II) socioeconomic status including education, income, and occupational status; (III) migration characteristics; (IV) public health and medical services including establishment of health records, health education, health insurance coverage, and hospitalization services; (V) family planning services.

### 2.2. Dependent Variables

The outcome of the analysis is the utilization of public health services, which is measured by two binary variables indicating the establishment of health records and the attendance of health education sessions in the city of residence, respectively. 

We are also interested in the content and methods of health education among internal migrants who received health education. As discussed in the introduction section, the prevention and control of NCD among internal migrants were often neglected. Thus, we are especially interested in whether migrants received any health education on NCD. We constructed a binary variable based on the question “whether migrants received health education content on NCD prevention, including nutrition health knowledge, chronic disease prevention, and mental health prevention.” In addition, the method of health education is measured by a binary variable indicating whether health education is done face-to-face or online. This variable is constructed based on the question “whether migrants received health education by the Internet.”

### 2.3. Independent Variables

The independent variables were socioeconomic, migration, and demographic characteristics.

#### 2.3.1. Socioeconomic Status

Variables included monthly income, educational attainment, employment status, and Hukou status. Income was measured as a continuous variable. Respondents’ education levels were categorized into four groups: primary school or below, middle school, high school, and college degree or above. Employment status contained three categories: unemployed, employed, and self-employed. Hukou status was categorized into two types: rural and urban Hukou. 

#### 2.3.2. Migration Characteristics

Migration characteristics were measured by the years of living in the city of residence, the range of migration, causes of migration, and where the migrants lived currently. Migrants may move across counties within a city, across cities within a province, or across provinces, which measured the range of migration. The causes of migration included seeking jobs, and following the migration of family members or other reasons. The place where the migrants lived was divided into two categories: urban and suburban areas.

#### 2.3.3. Demographic Characteristics

Gender, marital status (currently married vs. single), and age were included.

### 2.4. Statistical Analysis

We first described the characteristics of the respondents and their public health services utilization. The provincial variation of public health services utilization rates were then displayed by maps which were created by ArcGIS 9.3 (Esri, Redlands, CA, USA), a geographic information system for working with maps and geographic information. In addition, multivariate regressions were used to estimate the relationship between socioeconomic, migration, and demographic characteristics and public health services utilization. In order to control the homogeneity within each city, generalized multilevel models were used in multivariate regressions. All statistical analyses were conducted using SAS 9.4 (SAS Institute Inc., Cary, NC, USA) except for the maps. 

## 3. Results

### 3.1. Characteristics of the Respondents

The characteristics of study participants are presented in Table 1. Overall, the average age was around 35 years in both panels, and there were more males than females, with the majority married. Average monthly income increased from 3721 RMB in 2014 to 4077 RMB in 2015. About 85% of the respondents had completed the education of middle school or above and were registered as rural Hukou. In 2014, about 90% of internal migrants were either employed or self-employed, and this proportion dropped to 82% in 2015. These migrants had lived in the city of residence for around five years, about half migrated across provinces, and 70% lived in urban areas. According to the causes of migration, the percentage of those seeking jobs decreased slightly from 88.13% in 2014 to 84.39% in 2015. 

### 3.2. Utilization of Local Public Health Services among Internal Migrants

Table 2 demonstrates the utilization percentages of local public health services among internal migrants in China. In 2015, 29.10% of the migrants in the survey reported that they had established health records, which increased from 22.98% in 2014. The proportion of migrants who had accessed health education rose from 70.14% in 2014 to 90.70% in 2015. In terms of the content of health education, 95% of the migrants had accessed health education on infection diseases in both panels. Meanwhile, 80.86% of the migrants had accessed health education on NCD prevention in 2015, which increased from 66.54% in 2014. Almost all internal migrants accessed health education face-to-face in 2014 and 2015, whereas the proportion of internal migrants using the Internet increased from 43.04% in 2014 to 64.97% in 2015.

**Figure 1:**
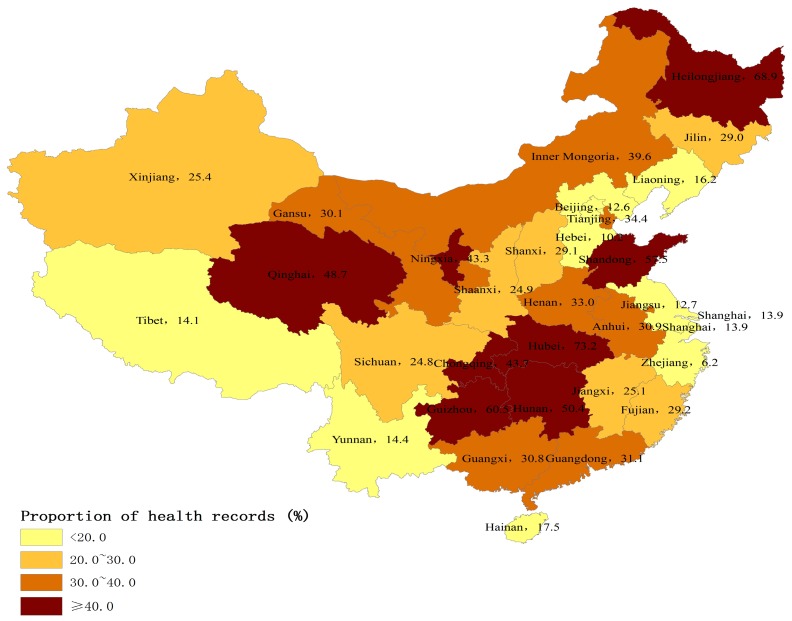
Percentage of internal migrants who had established health records by province, 2015 (%).

**Figure 2:**
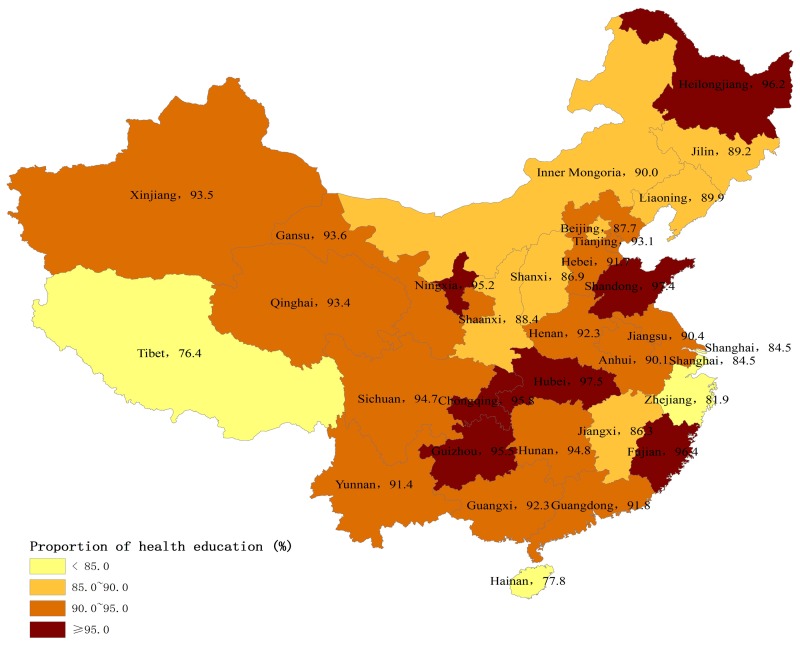
Percentage of internal migrants who had accessed health education by province, 2015 (%).

Figure 1 and Figure 2 show the provincial variation on the percentages of internal migrants who had established health records and accessed health education in 2015. The percentages of those using both public health services were higher in central China, and lower in eastern and western China. 

**Figure 3:**
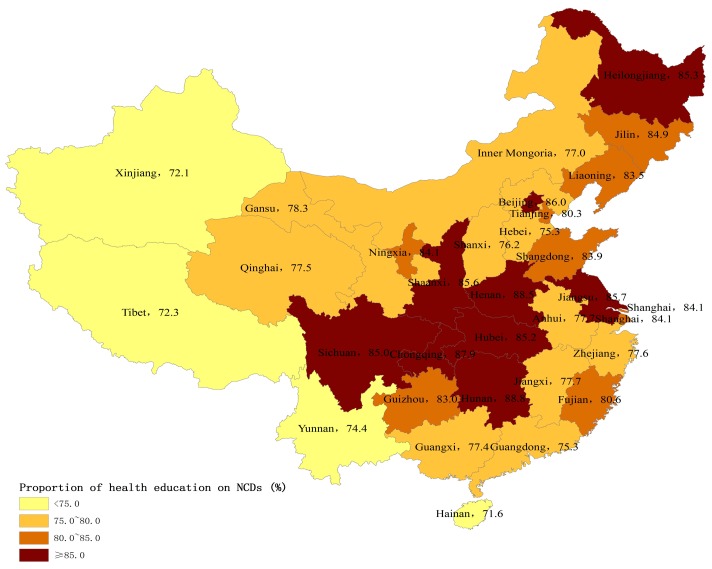
Percentage of internal migrants who had received health education on non-communicable diseases (NCD) prevention by province, 2015 (%).

**Figure 4:**
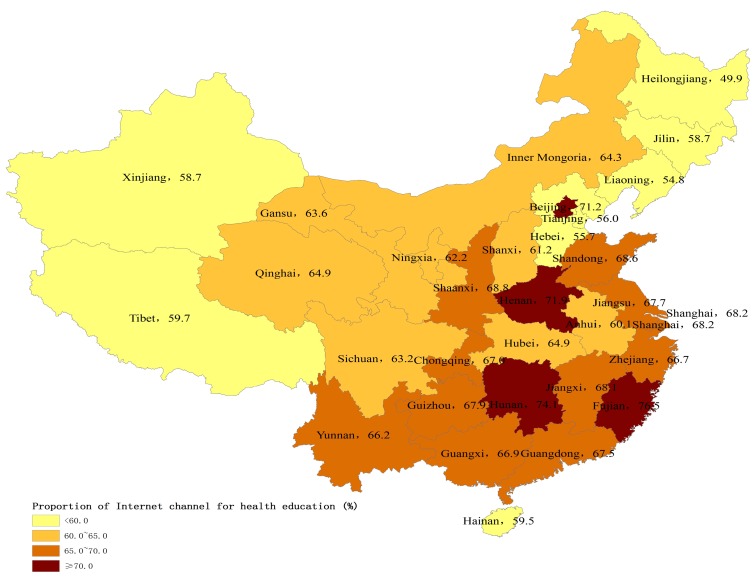
Percentage of internal migrants who had received health education through the Internet by province, 2015 (%).

Figure 3 and Figure 4 present the provincial variation on the percentages of internal migrants who received health education on NCD prevention and through the Internet in 2015. Internal migrants in western China were less likely to receive health education on NCD prevention and through the Internet than eastern and central China.

### 3.3. Determinants of Local Public Health Services Utilization among Internal Migrants

Table 3 presents the multivariate regression results for the factors associated with utilization of local public health services among all internal migrants (the second and third columns). In terms of the socioeconomic status, internal migrants with higher income and education levels were more likely to establish health records and access health education in their living cities. The internal migrants who had a college degree or above were more likely to establish health records (OR = 1.37, 95% CI: 1.32–1.42) and access health education (OR = 1.86, 95% CI: 1.78–1.94) than those with primary school or below. The employed or self-employed internal migrants had a significantly higher probability of using local public health services than those unemployed. It was also found that the internal migrants with urban Hukou were more likely to establish health records (OR = 1.09, 95% CI: 1.70–1.12) and access health education (OR = 1.06; 95% CI: 1.03–1.10).

With respect to the migration characteristics, the years of living in the city of residence was positively associated with the utilization of local public health services. Compared with internal migrants seeking jobs, those who had followed migrating family members or had other reasons for migration were more likely to establish health records (OR = 1.09, 95% CI: 1.05–1.13), but were less likely to access health education (OR = 0.96, 95% CI: 0.92–1.00). Compared to migration within a city, migration across provinces is negatively associated with the establishment of health records (OR = 0.88, 95% CI: 0.86–0.90) and utilization of health education (OR = 0.97, 95% CI: 0.94–1.00). Living in urban areas was more positively associated with the establishment of health records (OR = 1.26, 95% CI: 1.23–1.29) and utilization of health education (OR = 1.34, 95% CI: 1.31–1.37). 

In terms of the demographic characteristics, female and currently married internal migrants were more likely to establish health records and utilize health education. Increased age was related to a higher proportion of health records establishment but lower percentage of health education utilization. The probability of utilizing local public health services was much higher in 2015 than in 2014.

### 3.4. Determinants of the Content and Methods of Health Education among Internal Migrants Using Health Education

In Table 3, the multivariate regression results are reported for the factors associated with the content and methods of health education among internal migrants (the fourth and fifth columns). In terms of the socioeconomic status, income, education, and urban Hukou these factors positively affected access to health education on NCD or through the Internet. The internal migrants with college or higher levels of education were more likely to access health education on NCD (OR = 1.74, 95% CI: 1.67–1.81) and through the Internet (OR = 2.13, 95% CI: 2.06–2.21) than those with primary education or below. Compared to those unemployed, self-employed internal migrants had a higher likelihood of access to health education on NCD (OR = 1.12, 95% CI: 1.05–1.19) and through the Internet (OR = 1.13, 95% CI: 1.07–1.20). 

Regarding the migration characteristics, the longer the internal migrants lived in the city of residence, the higher the probability of accessing health education on NCD but there was no significant difference for usage of the Internet. The internal migrants who had followed migrating family members or had migrated for other reasons were more likely to utilize health education on NCD than those seeking jobs (OR = 1.10, 95% CI: 1.05–1.14), but there was no significant difference for usage of the Internet. Compared to migration within a city, migration across provinces and across cities was negatively associated with access to health education on NCD or through the Internet. Living in urban areas was positively associated with the access to health education on NCD (OR = 1.39, 95% CI: 1.36–1.42) and through the Internet (OR = 1.07, 95% CI: 1.05–1.09). 

In terms of the demographic characteristics, female and older migrants were more likely to receive health education on NCD, but were less likely to use the Internet for health education. Currently married participants were less likely to receive health education on NCD or through the Internet. 

## 4. Discussion

Using a nationally representative sample, this study sought to investigate the utilization of essential public health services and its determinants among internal migrants in China. Our study showed the great improvement of public health services coverage for internal migrants. Specifically, the coverage of health education increased by 20 percent, and the coverage of health education specifically on NCD prevention also increased by 14 percent between 2014 and 2015. More interestingly, there were remarkable regional differences in public health services utilization among internal migrants. Those internal migrants in more economically-rich municipalities and eastern coastal areas had lower utilization levels of public health services than those in central and northeast regions, which was inconsistent with their regional economic levels. Our study also found that migration characteristics and socioeconomic status were closely associated with the utilization of public health services among internal migrants, which has rarely been studied previously [10,16]. These improvements in health education coverage may be due to the government-subsidized national program, Equalization of Essential Public Health Services, and the subsidy increased year by year [9]. The government free-of-charge policy and subsidies may be extending the essential public health services to vulnerable populations such as internal migrants. It was notable that the coverage of health records only saw a slow increase. Our results indicated that only 29% of internal migrants established health records in 2015, whereas 75% of local residents had health records [19]. This finding was consistent with previous studies showing that internal migrants had less access to public health services [3,11]. In fact, health education uptake may not depend on their Hukou status, but establishment of health records was much more related with their Hukou status. Since the Hukou status is not easily transferred from rural to urban areas, internal migrants may face major obstacles to establishing health records and managing their health in cities [4].

There may be two reasons to explain the regional variations of public health services utilization. First, compared to local residents, the internal migrants may be more vulnerable in the more developed eastern areas than the other areas. In the developed eastern areas, there were much more factors that limited the interactions of internal migrants with local residents, leading to the larger gap between internal migrants and local residents. Previous research has showed that the social integration of internal migrants in the eastern areas was worse than in the other areas [20]. Second, the central government mainly provided subsidies to the underdeveloped central and western areas, and the delivery of public health services was considered to be the sole responsibility of local government in the developed eastern areas. According to the national policy, the central government should provide 80% and 60% of total subsidies to the western and central regions to deliver essential public health services, respectively, but only 10–50% to the eastern regions [21]. For example, Gansu province, a less-developed region, received the subsidy from the central government, but Zhejiang province, a developed region, did not receive any subsidy [22,23]. The subsidies from the central government may be more likely to target internal migrants than those from local governments. 

Migration characteristics might also influence public health services utilization in different ways. First, migration would reduce the continuum and compliance with public health services. Because of residential changes and job instabilities, internal migrants had little incentive to establish health records in the city where they lived, and our study indicated that less than 30% of internal migrants established health records in the city in 2015. Lack of health records brought challenges to overall health management. Instead, cross-regional resource and information sharing would improve the continuum of public health services for internal migrants [16]. Second, internal migrants may face more stress when they hunt for jobs, and find and purchase housing and child education [13], and may have less time to participate in health education. Previous research also reported that encouragement of internal migrants to participate in community activities was important to improve the accessibility of public health services [10]. Third, we found that years of migration would increase the probability of utilizing public health services and accessing health education on NCD or through the Internet, whereas range of migration would result in a decrease. Therefore, internal migrants who moved to the city of residence for a shorter time period or migrated across provinces were more vulnerable and should be given more attention when providing essential public health services.

In addition, public health services utilization for internal migrants saw a large disparity by socioeconomic status. Higher education level, the employed, and urban Hukou were the main protective factors for public health services utilization among internal migrants, which are consistent with previous studies [10,17]. The internal migrants with higher education levels usually have better knowledge and awareness about public health services [10]. The employed internal migrants with formal jobs were more likely to establish health records and utilize health education about occupation diseases and NCD in their community. The internal migrants with urban Hukou had equal access to public health services as local residents [4], indicating that rural Hukou was a key barrier to accessing public health services for internal migrants. Therefore, internal migrants with low socioeconomic status should be given more attention in urban healthcare systems in order to achieve the equalization of public health services.

This study has several limitations. First, the public health services policies for internal migrants in each city were not available, and we cannot identify the effect of different policies by city. To control for the effects of city-level variables, we included the city dummy variables in the analysis. Second, internal migrants with poor health may be more likely to use public health services, but we had no data on their health status. Since age is the key predictor of health, we included age in our analysis. Third, the indicators—health records and health education—do not represent the full package of the essential public health services, and they were just a part of the full package. However, in this survey, only information on health records and health education were collected, and no information on other public health services indicators were available.

## 5. Conclusions

Public health services coverage for internal migrants has seen great improvement due to government subsidies. Internal migrants with lower socioeconomic status and who have migrated across provinces are less likely to use public health services and need to be targeted. More attention should be given to the local government in the developed eastern regions in order to narrow the regional gaps and to achieve the equalization of public health services.

## Figures and Tables

**Table 1:** Characteristics of the respondents in National Internal Migrants Dynamic Monitoring Survey (NIMDMS), 2014 and 2015.

Characteristics	2014, N (%)	2015, N (%)
*Demographic characteristics*		
Gender		
Male	117,624 (58.55)	109,300 (53.06)
Female	83,273 (41.45)	96,689 (46.94)
Age (year) *	34.66 (9.40)	36.29 (10.64)
Marital status		
Single	47,997 (23.89)	43,521 (21.13)
Married	152,899 (76.11)	162,468 (78.87)
*Socioeconomic status*		
Monthly income (RMB) *	3721.86 (5439.44)	4077.82 (4873.15)
Education		
Primary school or below	27,911 (13.89)	31,336 (15.21)
Middle school	105,874 (52.70)	104,005 (50.49)
High school	41,280 (20.55)	44,774 (21.74)
College degree or above	25,832 (12.86)	25,874 (12.56)
Employment status		
Unemployed	24,537 (12.21)	36,443 (17.69)
Employed	82,099 (40.87)	78,902 (38.30)
Self-employed	94,257 (46.92)	90,641 (44.00)
Hukou status		
Rural Hukou	170,870 (85.05)	174,691 (84.81)
Urban Hukou	30,027 (14.95)	31,298 (15.19)
*Migration characteristics*		
Years of living in the city of residence *	5.06 (4.78)	5.17 (4.95)
Causes of migration		
Seeking jobs	177,045 (88.13)	173,829 (84.39)
Family members following migrants or other reasons	23,852 (11.87)	32,160 (15.61)
Migration range		
Across provinces	102,371 (50.96)	102,781 (49.90)
Across cities within a province	60,933 (30.33)	62,503 (30.34)
Across counties within a city	37,593 (18.71)	40,705 (19.76)
Currently living area		
Urban	140,460 (69.92)	146,691 (71.21)
Suburban	60,437 (30.08)	59,298 (28.79)
N	200,937	206,000

Note: * Mean, SD. RMB: renminbi.

**Table 2:** Utilization of local public health services among internal migrants in NIMDMS, 2014 and 2015.

Public Health Services	2014 (%)	2015 (%)
Establishment of health records	22.98	29.10
Accessing health education	70.14	90.70
Content of health education accessed		
(1) Prevention of infectious diseases	95.98	94.38
(2) Prevention of non-communicable diseases	66.54	80.86
Methods for accessing health education		
(1) Face-to-face	99.26	99.12
(2) Internet	43.04	64.97

**Table 3:** Determinants of local public health services utilization, the contents and channels of health education.

Variables	Total Sample		Subsample Using Health Education	
	Establishment of Health Records	Using Health Education	NCD Content	Internet Method
**Socioeconomic status**				
Monthly income (1000 RMB)	1.00 (1.00–1.01) **	1.01 (1.00–1.01) ***	1.01 (1.01–1.01) ***	1.02 (1.02–1.02) ***
Education (referred to primary school or below)				
Middle school	1.17 (1.13–1.20) ***	1.34 (1.30–1.38) ***	1.16 (1.12–1.19) ***	1.44 (1.40–1.48) ***
High school	1.24 (1.20–1.28) ***	1.58 (1.52–1.63) ***	1.34 (1.30–1.39) ***	1.72 (1.67–1.77) ***
College degree or above	1.37 (1.32–1.42) ***	1.86 (1.78–1.94) ***	1.74 (1.67–1.81) ***	2.13 (2.06–2.21) ***
Employment status (referred to unemployed)				
Employed	1.16 (1.01–1.24) ***	1.26 (1.18–1.35) ***	1.01 (0.94–1.07)	1.19 (1.12–1.26) ***
Self-employed	1.11 (1.04–1.18) **	1.17 (1.09–1.25) ***	1.12 (1.05–1.19) ***	1.13 (1.07–1.20) ***
Urban Hukou	1.09 (1.07–1.12) ***	1.06 (1.03–1.10) ***	1.12 (1.09–1.15) ***	1.13 (1.10–1.16) ***
**Migration characteristics**				
Years of living in the city of residence	1.02 (1.02–1.02) ***	1.01 (1.00–1.01) ***	1.01 (1.01–1.01) ***	1.00 (1.00–1.00)
Causes of migration (referred to seeking jobs)				
Family members following migrants or other reasons	1.09 (1.05–1.13) ***	0.96 (0.92–1.00) *	1.10 (1.05–1.14) ***	0.97 (0.94–1.00)
Migration range (referred to across counties within a city)				
Across provinces	0.88 (0.86–0.90) ***	0.97 (0.94–1.00) *	0.92 (0.89–0.95) ***	0.96 (0.94–0.99) **
Across cities within a province	0.98 (0.96–0.90)	1.00 (0.97–1.04)	0.96 (0.93–0.98) **	0.92 (0.89–0.94) ***
Living in urban areas	1.26 (1.23–1.29) ***	1.34 (1.31–1.37) ***	1.39 (1.36–1.42) ***	1.07 (1.05–1.09) ***
**Demographic characteristics**				
Female	1.14 (1.12–1.16) ***	1.30 (1.27–1.32) ***	1.08 (1.06–1.10) ***	0.90 (0.89–0.92) ***
Age (10 years)	1.01 (1.00–1.02) *	0.92 (0.91–0.93) ***	1.05 (1.04–1.06) ***	0.73 (0.72–0.74) ***
Currently married	1.35 (1.32–1.39) ***	1.47 (1.44–1.51) ***	0.88 (0.86–0.90) ***	0.97 (0.95–0.99) *
2015	1.55 (1.53–1.58) ***	4.88 (4.78–4.98) ***	2.27 (2.23–2.31) ***	2.99 (2.94–3.04) ***
Observations	352945	352945	284490	284488

Notes: Odds ratio and 95% confidence intervals were shown. Significance level: *** *p* < 0.001, ** *p* < 0.01, * *p* < 0.05.
